# 

**Published:** 1887-07-02

**Authors:** 


### THE HOSPITAL

An Institution, Family, and Parochial Journal of Hospitals, Asylums, & all Agencies for the Care of the Sick, Criticism & News.

### CONTENTS.

**Two Guineas, Madam** - - - - - - 225
**Words of Consolation.**—XXXIX. The Work of the Holy Ghost - - - - - - 226
**Flowers for the London Poor** - - - 226
**Counterparts.** By a Student of Eastern Philosophies.—Chap. IV. A Discovery - - - 227
**The National Pension Fund** - - - 228
**Questions and Answers** - - - - 228
**A Noble Profession: Nursing the Sick.**—The Nurse and the Sick-room. By Miss Mollett - 229
**Patients:** from the Doctor's Point of View. By a Consulting Physician - - - - - 231
**Notes and News.**—Hospital Appointments.—Working Men and the Hospitals.—Vacancies. —Photographs and Albums for Hospitals. — The Princess Alice Hospital Jubilee Fête.—State-supported Hospitals.—Church Parade in Aid of the Children's Hospital.—Hospital Collection-Boxes in Theatres.—Distribution of the Sunday Fund Number of The Hospital.—The Dundee Royal Infirmary, etc. - - - - 232
**Annotations.**—Careless Dispensing.— The Growth of the Sunday Fund.—Doctors "out in the cold" - - 235
**Unlicensed Practitioners** - - - - 236
**The Out-Patient Department.**—No. II. By Mr. Burdett, Miss Twining, Professor Tweedy, Mr. Michelli, Dr. Collier, and Sir Andrew Clark - 236
**Are there more Lunatics than formerly?** By Dr. Hack Tuke - - - - - 239
**Till the Doctor Comes.**—XXI. Treatment of Emergencies - - - - - - - 239
**The Children's Corner.**—Puzzles, Answers, etc. - 240
**Amusements with Profit** - - - - 240

